# Use of Caripracina in a case of manic episode with psychotic symptoms difficult to treat due to side effects with the use of psychotropic medication. A case report

**DOI:** 10.1192/j.eurpsy.2023.1483

**Published:** 2023-07-19

**Authors:** P. Casado De La Torre

**Affiliations:** Psychiatry, Sistema Andaluz de Salud (SAS), Algeciras, Spain

## Abstract

**Introduction:**

There are manic episodes to involve a challenge in treatment due to finding resistance or secondary effects with the drugs of choice, this situation oblige forcing us to seek alternatives in the data sheet.

**Objectives:**

To describe the complicated evolution of a case of acute mania difficult to threat with stabilizer drugs and antipsychotics of choice. We discuss the psychopharmacological approach.

**Methods:**

Case summary. We have conduced a systematic review of the descriptions published to date, regarding this case.We presented a case, in a 48-year-old female, admitted to our hospital due to psychopathological descompensation of bipolar affective disorder, where we observed manic and psychotic symtoms.

**Results:**

In the first instance, we started treatment with Lithium and Olanzapine, in increasing doses, along with benzodiazepine support.

During more than four months of follow-up , multiple drugs have been tested sequentially: olanzapine, aripiprazole and quetiapine. We observed a good response but low tolerance issue to secondary effects consisting of severe akathisia, in progressive stiffness (spasticity) and hands tremor, it was very disabling problem for patient, even though the use of biperiden.

This situation forced us to search another option of treatment, different from non-pharmacological therapies (ECT). After checking the literature and publications about it, we decided to start treatment with Caripracine 3mg/24h, for which the therapeutic indication is the treatment of manic with mixed symtoms.

**Conclusions:**

We propose, through a clinical case, the use of cariprazine as a first choice in the acute decompensation of bipolar affective disorder, without symptoms of mixed mania.

During the treatment, the patient presente multiple difficulties and finally, a good response is was obtained with the use of Cariprazine, althought this patient continued showing slight akathisia well tolerated, she was able to leave after four months of hospitalization in the acude mental health unit.

**Disclosure of Interest:**

None Declared

